# Performance of the Manchester triage system in older emergency department patients: a retrospective cohort study

**DOI:** 10.1186/s12873-018-0217-y

**Published:** 2019-01-07

**Authors:** Steffie H. A. Brouns, Lisette Mignot-Evers, Floor Derkx, Suze L. Lambooij, Jeanne P. Dieleman, Harm R. Haak

**Affiliations:** 1 Department of Internal Medicine, Máxima Medical Centre, 5600 BM Eindhoven/Veldhoven, the Netherlands; 2 Department of Health Services Research, and CAPHRI School for Public Health and Primary Care, Maastricht University, 6229 ER Maastricht, the Netherlands; 3 Department of Emergency medicine, Máxima Medical Centre, 5600 BM Veldhoven, the Netherlands; 4 Máxima Medical Centre Academy, Máxima Medical Centre, Eindhoven/Veldhoven, the Netherlands; 5 Department of Internal Medicine, Division of general medicine, Maastricht University Medical Centre, 6229 HX Maastricht, the Netherlands

**Keywords:** Emergency services hospital, Triage, Aged, Outcome assessment (health care)

## Abstract

**Background:**

Studies on the reliability of the MTS and its predictive power for hospitalisation and mortality in the older population have demonstrated mixed results. The objective is to evaluate the performance of the Manchester Triage System (MTS) in older patients (≥65 years) by assessing the predictive ability of the MTS for emergency department resource utilisation, emergency department length of stay (ED-LOS), hospitalisation, and in-hospital mortality rate. The secondary goal was to evaluate the performance of the MTS in older surgical versus medical patients.

**Methods:**

A retrospective cohort study was conducted of all emergency department visits by patients ≥65 years between 01 and 09-2011 and 31-08-2012. Performance of the MTS was assessed by comparing the association of the MTS with emergency department resource utilisation, ED-LOS, hospital admission, and in-hospital mortality in older patients and the reference group (18–64 years), and by estimating the area under the receiver operating characteristics curves.

**Results:**

Data on 7108 emergency department visits by older patients and 13,767 emergency department visits by patients aged 18–64 years were included. In both patient groups, a higher emergency department resource utilisation was associated with a higher MTS urgency. The AUC for the MTS and hospitalisation was 0.74 (95%CI 0.73–0.75) in older patients and 0.76 (95%CI 0.76–0.77) in patients aged 18–64 years. Comparison of the predictive ability of the MTS for in-hospital mortality in older patients with patients aged 18–64 years revealed an AUC of 0.71 (95%CI 0.68–0.74) versus 0.79 (95%CI 0.72–0.85). The majority of older patients (54.8%) were evaluated by a medical specialty and 45.2% by a surgical specialty. The predictive ability of the MTS for hospitalisation and in-hospital mortality was higher in older surgical patients than in medical patients (AUC 0.74, 95%CI 0.72–0.76 and 0.74, 95%CI 0.68–0.81 versus 0.69, 95%CI 0.67–0.71 and 0.66, 95%CI 0.62–0.69).

**Conclusion:**

The performance of the MTS appeared inferior in older patients than younger patients, illustrated by a worse predictive ability of the MTS for in-hospital mortality in older patients. The MTS demonstrated a better performance in older surgical patients than older medical patients regarding hospitalisation and in-hospital mortality.

**Electronic supplementary material:**

The online version of this article (10.1186/s12873-018-0217-y) contains supplementary material, which is available to authorized users.

## Background

Over the past few decades the number of Emergency Department (ED) visits has increased substantially, resulting in ED crowding [1, 2]. This leads to prolonged ED length of stay (ED-LOS), treatment delay and reduced patient satisfaction, these are associated with adverse patient outcomes and a longer hospital stay [3–6]. In particular, the ageing of the population has a major impact on emergency care. Older patients (≥ 65 years old) presently account for up to 30% of all ED visits, which is expected to increase further [2, 7–9].

Older patients more often than younger patients present with atypical signs, symptoms and multi-morbidity, they therefore represent a complex population at the ED [10, 11]. EDs may not be appropriately suited to these circumstances, as emergency care is focused on rapid assessment and treatment of acutely ill patients rather than addressing complex medical and social problems [12]. Consequently, older patients at the ED have a higher risk of being misdiagnosed than younger patients, potentially resulting in inadequate treatment and a poor outcome [11].

EDs require a valid and reliable triage system to rapidly prioritise patients presenting in the ED based on their clinical urgency, as to efficiently plan available resources and time. Since 2003 the Dutch EDs use the five-level Manchester Triage System (MTS) to determine treatment priority [13]. Studies on the reliability of the MTS and its predictive power for hospitalisation and mortality in the general and paediatric population, however, have demonstrated mixed results [14–20]. Furthermore, the performance of the MTS differs between medical versus surgical specialties [14]. There is also some evidence suggesting that the MTS performs worse in the older population [21, 22].

The objective of this study was to evaluate the performance of the MTS in older patients (≥65 years) by assessing the predictive ability of the MTS for ED resource utilisation, ED-LOS, hospitalisation, and in-hospital mortality. The secondary objective was to compare the performance properties of the MTS between older surgical patients and older medical patients.

## Methods

### Study design, setting and participants

A retrospective cohort study was conducted at a teaching hospital in the Netherlands [23]. Approximately 28,000 patients visit the ED annually, of which about 25% are patients aged 65 years and older. Primary healthcare is accessible for every citizen 24 h a day and provides an important safety net in the Netherlands. Emergency departments cooperate closely with general practitioners (GPs) and the ambulance services. Patients are predominantly referred by a GP in the Dutch acute care system. Other modes of referral are by self-referral, referral by a medical specialist or ambulance [24]. In addition, in case of referral by GPs, they will provide the primary assessment for which medical specialty the patient will be referred. Assessment of patients presenting to the ED is predominantly performed by a non-trainee resident, a trainee resident or an emergency physician, supervised by a medical specialist [25, 26].

Data on all consecutive ED visits between September 1st 2011 and August 31st 2012 were extracted from electronic patient records by one investigator using a standard data collection form. Multiple visits per patient were possible and numbered accordingly in the database. Patients referred for cardiology are predominantly cared for in the emergency cardiac care unit, and therefore are not part of this study. Additionally, the majority of patients with gynaecologic and obstetric emergencies are cared for elsewhere in the hospital and do not present to the ED. Exclusion criteria were patients aged < 18 years old, visits with a missing triage level and patients directly transferred to another department. Patients aged 18–64 years were included as a reference group to the older patients. Exemption of ethical approval by the Institutional Review Board of Máxima Medical Centre was acquired.

### Manchester triage system

Triage at the ED presentation is performed using the MTS [13]. This five-level system, developed by a consensus group in the United Kingdom, is based on 52 flowcharts representing pre-defined symptoms, such as “shortness of breath” and “abdominal pain”. In addition, each flowchart comprises of six key discriminators, such as danger to life, or severe pain, in order to distinguish between urgency categories [13]. The MTS consists of the following urgency categories corresponding to the maximum waiting time for first contact with a physician: 1. immediate (red), 2. very urgent (orange), evaluation within 10 min, 3. urgent (yellow), evaluation within 60 min, 4. standard (green), evaluation within 120 min and 5. non-urgent (blue), evaluation within 240 min [13]. The non-urgent level (blue) is not being used at our ED. All triage nurses are specialised ED nurses and have received specific training in applying the MTS using a computerised triage programme. Triage is performed either in the dedicated triage room or in a treatment room, when patients arrive by ambulance.

### Data collection and definitions

The primary outcome of interest was the performance of the MTS as assessed by the predictive ability for ED resource utilisation, ED-LOS, hospitalisation and in-hospital mortality of older ED patients (aged ≥65 years old). ED resource utilisation comprised of the number of diagnostic tests, medical procedures, medication administration and the number of specialty consultations. Diagnostic tests performed on the ED consisted of laboratory tests, urine tests, cultures, electrocardiograms, X-rays, ultrasonography, computed tomography scans, and magnetic resonance imaging. Placement of intravenous access, intubation, placement of urinary catheter or gastric tube, cardiac rhythm monitoring, wound, eye or compressive bandage, plaster cast, sling, and tetanus vaccination were considered as medical procedures. Final disposition was categorised into discharge home without follow-up, discharge home with follow-up by a GP or in an outpatient clinic, admission to the acute medical unit, admission to a high care unit, or admission to another hospital ward, died on ED and left without been seen by a physician (LWBS). Intensive care unit (ICU), medium care unit (MCU), stroke care unit (SCU) and cardiac care unit (CCU) were considered high care units. Data on admission to a high care unit was extracted manually from electronic patient records and only collected for the older patients. The primary medical specialty involved on the ED was divided into surgical (including general surgery, plastic surgery, urology, orthopaedics, otorhinolaryngology, ophthalmology, dermatology, oral surgery and gynaecology) and medical (including internal medicine, pulmonology, cardiology, neurology, psychiatry, gastroenterology and rheumatology). The time of presentation was divided into day (8 am – 5 pm), evening (5 pm – 12 pm) and night (12 pm – 8 am). The mode of referral was categorised as referral by GP, ambulance or medical specialist and self-referral. ED recording times (in minutes) were sectioned into 1) time in waiting room: time from ED arrival to ED bed placement, 2) treatment time: time from ED bed placement to end treatment time and 3) ED-LOS: time between ED arrival and ED discharge or hospital admission [24].

### Statistical analysis

Statistical analyses were performed using SPSS (IBM SPSS Statistics for Windows, version 22.0, Armonk, New York). The unit of analysis for this study was the ED visit thereby assuming independence of multiple visits by the same patient. Comparisons of the patient characteristics per triage category were tested using the Chi-square test for categorical variables. The Mann-Whitney U test, the Kruskal-Wallis test, the T-test, or ANOVA (analysis of variance) were used for the comparison of continuous variables depending on the number of groups and distribution. Continuous variables were described by means of standard deviation (SD) or medians with interquartile range (IQR) as appropriate.

The association between the MTS category and dichotomous outcome variables was described by odds ratios (OR) and corresponding 95% confidence intervals (CI) as calculated by an univariable logistic regression analysis. The association between MTS category and ED-LOS was expressed as a regression coefficient and the corresponding 95% CI as calculated using linear regression analysis. A poisson regression analysis was performed to assess the association between the MTS category and the number of diagnostic tests and medical procedures performed. Results were expressed as incidence density ratios (IDR) with a 95% CI. The MTS category green was considered as the reference category in the regression analyses. Multivariable regression analysis was not performed as we were interested in the predictive ability of the MTS only. Patients that died on the ED were considered as not admitted to the hospital and excluded from the analyses on admission, and in-hospital mortality.

To compare the predictive ability of the MTS between older patients and the reference group (i.e. aged 18–64 years), we performed regression analyses with the MTS category as the independent variable stratified by age category. In addition, to compare the performance of the MTS in older patients assessed by a surgical versus medical specialty, a regression analysis was used stratified by the primary specialty on the ED.

The ability of the MTS categories to predict hospitalisation, admission to a high care unit, and in-hospital mortality was evaluated by estimating the area under the receiver operating characteristics (ROC) curves. A higher area under the curve (AUC) indicates a better accuracy; an AUC of 1.0 indicates an excellent performance and an AUC of 0.5 indicates a 50% chance of an accurate score. A two-sided *p*-value < 0.05 was considered significant. The Bonferroni test was performed to correct for multiple comparisons. A p-value < 0.0036 was considered significant after Bonferroni correction.

## Results

**Fig. 1:**
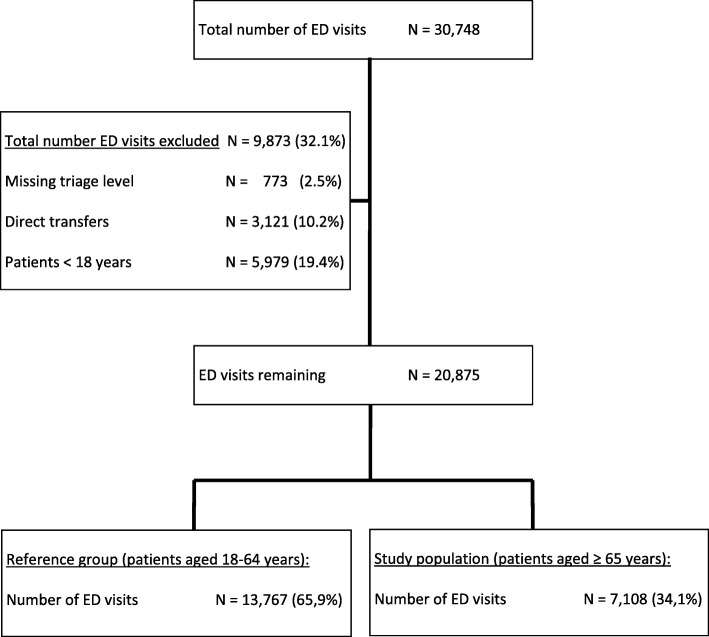
Flow chart of the studied population. ED = emergency department. MTS = Manchester Triage System

During the study period, 30,748 ED visits were recorded. In total, 9873 (32.1%) ED visits were excluded because they consisted of a paediatric population (19.4%), a missing triage level (2.5%) or direct transfers to other departments (10.2%). Older patients (aged ≥65 years) accounted for 34.1% (*n* = 7108) out of 20,875 eligible ED visits and were considered for the study population. In addition, 13,767 ED visits by patients aged 18–64 years old were included as the reference population (Fig. 1).

The majority of older patients were assigned to the MTS category yellow (44.5%), followed by green (39.7%), orange (15.0%), and red (0.9%). In the group aged 18–64 years old, patients were predominantly assigned to the MTS category green (59.7%), followed by yellow (32.0%), orange (7.9%) and red (0.4%) (*p* < 0.001 compared with older patients).

### Patient characteristics

The mean age of the entire older study population was 77.0 years (SD 7.6 years). 45.8% were male compared with 55.6% males in the group aged 18–64 years (*p* < 0.001). In both groups, male patients had higher urgency levels than female patients. The primary mode of referral for ED visits by older patients was by GP (57.5%), especially in categories orange, yellow and green (61.7, 63.4, and 49.5% respectively, *p* < 0.001). In category red, both older patients and patients aged 18–64 years predominantly arrived by ambulance (55.7 and 60%). In 3215 ED visits (45.2%), older patients were primarily treated by a surgical specialty and in 3893 ED visits (54.8%) by medical specialties (Table 1). Patients aged 18–64 years were predominantly treated by a surgical specialty (70.5%) (Additional file 1: Table S1).

**Table 1:** Characteristics of emergency department visits by older patients per MTS category

	MTS category			
	Red (*n* = 61)	Orange (*n* = 1063)	Yellow (*n* = 3165)	Green (*n* = 2819)
Mean age in years (SD)**	77.0 (6.9)	77.3 (7.3)	77.4 (7.7)	76.4 (7.7)
Male participants (%)**	34 (55.7%)	575 (54.1%)	1430 (45.2%)	1215 (43.1%)
Time of presentation (%)**				
- Day	30 (49.2%)	612 (57.6%)	2021 (63.9%)	1937 (68.7%)
- Evening	17 (27.9%)	300 (28.2%)	874 (27.6%)	757 (26.9%)
- Night	14 (23.0%)	151 (14.2%)	270 (8.5%)	125 (4.4%)
Mode of referral (%)**				
- General practitioner	24 (39.3%)	639 (61.7%)	1909 (63.4%)	1297 (49.5%)
- Self-referral	2 (3.3%)	85 (8.2%)	302 (10.0%)	652 (24.9%)
- Ambulance	34 (55.7%)	237 (22.9%)	464 (15.4%)	177 (6.8%)
- Medical specialist	1 (1.6%)	75 (7.2%)	338 (11.2%)	493 (18.8%)
Medical specialty (%)**				
- Surgical	9 (14.8%)	160 (15.1%)	1271 (40.2%)	1775 (63.0%)
- Medical	52 (85.2%)	903 (84.9%)	1894 (59.8%)	1044 (37.0%)

*MTS* Manchester Triage System; *SD* Standard Deviation; *ED* Emergency Department; Surgical includes: general surgery, plastic surgery, urology, orthopaedics, otorhinolaryngology, ophthalmology, dermatology, oral surgery, gynaecology; Medical includes: internal medicine, pulmonology, cardiology, neurology, psychiatry, gastroenterology, rheumatology;

*P*-values were calculated using ANOVA and Chi-square test; ** = *p* < 0.001

### ED recording times

The median time in the waiting room was 3 min (IQR 0–11 min) in older patients and 7 min in patients aged 18–64 years (IQR 2–24 min) (*p* < 0.001). In both groups, the time in the waiting room increased as the MTS urgency decreased, with the longest time in category green (in older patient 5 min, IQR 0–23, and in patients aged 18–64 years 10 min, IQR 4–34) (*p* < 0.001). Overall, the median ED-LOS was 136 min (IQR 94–180) in older patients, and 99 min (IQR 61–146) in patients aged 18–64 years (*p* < 0.001). In ED visits by older patients, the median ED-LOS was longest in category yellow (147 min, IQR 109–189), while the median ED-LOS was longest in category orange in patients aged 18–64 years (127 min, IQR 94–170) (Table 2 and Additional file 1: Table S1).

**Table 2:** ED-LOS, ED resource utilisation, hospitalisation and in-hospital mortality per MTS category in older patients

	MTS category			
	Red (n = 61)	Orange (n = 1063)	Yellow (n = 3165)	Green (*n* = 2819)
Median ED-LOS in minutes (IQR)**	105 (59–139)	139 (104–179)	147 (109–189)	120 (75–168)
Number of diagnostic tests**				
- mean (SD)	3.1 (2.1)	3.7 (1.8)	2.5 (1.7)	1.3 (1.3)
- none (%)	11 (18.0%)	30 (2.8%)	345 (10.9%)	952 (33.8%)
Number of medical procedures**				
- mean (SD)	3.4 (1.7)	1.5 (1.1)	1.5 (1.1)	0.9 (0.9)
- none (%)	6 (9.8%)	44 (4.1%)	643 (20.3%)	1166 (41.4%)
Medication administered at the ED**	40 (65.6%)	699 (65.8%)	1518 (48.1%)	735 (26.1%)
> 1 specialty consultations at ED**	8 (13.1%)	225 (21.2%)	553 (17.5%)	234 (8.3%)
Disposition				
- Discharge home (%)	–	29 (2.7%)	307 (9.7%)	589 (20.9%)
- Discharge home + follow-up (%)	–	54 (5.1%)	613 (19.4%)	1278 (45.3%)
- Admission acute medical unit (%)	6 (9.8%)	663 (62.4%)	1883 (59.5%)	874 (31.0%)
- Admission high care unit (%)	35 (57.4%)	228 (21.4%)	211 (6.7%)	19 (0.7%)
- Admission other hospital ward (%)	9 (14.8%)	87 (8.2%)	150 (4.7%)	58 (2.1%)
- LWBS (%)	–	–	–	1 (0.0%)
- Died in ED (%)	11 (18.0%)	2 (0.2%)	1 (0.0%)	–
In-hospital mortality^#^ (%)	17 (34.0%)	132 (12.5%)	122 (3.9%)	47 (1.7%)

*MTS* Manchester Triage System; *SD* Standard Deviation; *ED* Emergency Department; *IQR* interquartile range; *ED-LOS* emergency department length of stay; High care unit = intensive care unit, medium care unit, stroke care unit and cardiac care unit; *LWBS* left without being seen by a physician; *P*-values were calculated using ANOVA, Kruskal-Wallis test and Chi-square test; ** = *p* < 0.001; # = patients that have died in the ED are excluded from this analysis

Comparison of the association between the ED-LOS and the MTS category in older patients versus patients aged 18–64 years revealed a better association of the ED-LOS and the MTS category in older patients than in patients aged 18–64 years (Table 3).

**Table 3:** Association between outcome measures and MTS category in older patients and patients aged 18–64 years

Outcome measure	Group	MTS category – relative estimates with 95% confidence intervals			
		Red^‡^ (n = 61)	Orange^‡^ (n = 1063)	Yellow^‡^ (n = 3165)	Absolute value in green reference category (n = 2819)
ED-LOS (minutes, difference)^¶^	18–64 years	36 (18.9–53.1)**	42.9 (38.8–47.0)**	41.3 (38.9–43.7)**	Median 80 (IQR 50–124)
	≥ 65 years	−17 (−34.7–0.4)	20.1 (15.3–25.0)**	28.8 (25.3–32.3)**	Median 120 (IQR 75–168)
Number of diagnostic tests (IDR)^	18–64 years	4.6 (4.0–5.3)**	3.7 (3.6–3.9)**	2.4 (2.3–2.5)**	Mean 0.7 (SD 0.9)
	≥ 65 years	2.4 (2.1–2.8)**	2.9 (2.7–3.0)**	1.9 (1.8–2.0)**	Mean 1.3 (SD 1.3)
Number of medical procedure (IDR)^	18–64 years	4.8 (4.2–5.5)**	2.6 (2.5–2.8)**	1.3 (1.2–1.3)**	Mean 0.8 (SD 0,8)
	≥ 65 years	3.8 (3.3–4.4)**	2.9 (2.7–3.0)**	1.7 (1.6–1.7)**	Mean 0.9 (SD 0.9)
Medication administered (OR)^±^	18–64 years	21.4 (10.1–45.3)**	5.2 (4.6–6.0)**	3.8 (3.5–4.1)**	*N* = 1800 (21.9%)
	≥ 65 years	5.4 (4.7–6.3)**	5.4 (4.7–6.3)**	2.6 (2.4–2.9)**	*N* = 735 (26.1%)
> 1 specialty consultation (OR)^±^	18–64 years	13.2 (7.3–23.9)**	8.1 (6.6–9.8)**	4.2 (3.6–5.0)**	*N* = 242 (2.9%)
	≥ 65 years	1.7 (0.8–3.5)	3.0 (2.4–3.6)**	2.3 (2.0–2.8)**	*N* = 234 (8.3%)
Hospitalisation (OR) ^#±^	18–64 years	74.9 (31.8–176.1)**	19.2 (16.6–22.3)**	7.0 (6.4–7.8)**	*N* = 757 (9.3%)
	≥ 65 years	47.4 (11.5–195.3)**	19.4 (15.5–24.2)**	4.8 (4.3–5.3)**	*N* = 951 (33.7%)
In-hospital mortality (OR)^#±^	18–64 years	190.9 (68.5–531.8)**	15.4 (6.6–36.0)**	5.1 (2.3–11.6)**	*N* = 8 (0.1%)
	≥ 65 years	29.7 (15.5–57.0)**	8.2 (5.9–11.5)**	2.3 (1.7–3.3)**	*N* = 47 (1.7%)

*MTS* Manchester Triage System; *ED* Emergency Department; *ED-LOS* emergency department length of stay; *IQR* inter quartile range; *SD* standard deviation; *IDR* incidence density ratio; *OR* odds ratio; ‡ = Comparison of MTS category in both older patients and patients 18–64 years with reference category, which is MTS category green; ¶ = difference with MTS green in minutes, analysed with linear regression, reporting regression coefficients and 95% confidence intervals; ^= analysed with Poisson regression, reporting incidence density ratios; ± = analysed with logistic regression, reporting odds ratio and 95% confidence intervals; # patients who have died in the ED are excluded from the analyses; ** = *p* < 0.001

### ED resource utilisation

Overall, the mean number of diagnostic tests performed on the ED was 2.2 (SD 1.8) in older patients and 1.2 (SD 1.3) in the reference group (*p* < 0.001). In older patients, the mean number of medical procedures on the ED was 1.4 (SD 2.2) compared with 1.0 (SD 1.0) in patients aged 18–64 years (*p* < 0.001). Older patients assigned to category orange received the highest number of diagnostic tests (mean 3.7, SD 1.8) (Table 2). In patients aged 18–64 years, both the mean number of diagnostic tests and the mean number of medical procedures decreased as the MTS urgency decreased (Additional file 1: Table S1). Furthermore, in 14.4% of the ED visits by older patients and in 7.1% of the ED visits by patients 18–64 years, > 1 specialty consultations took place on the ED. Category orange in older patients (21.2%) and category red in patients 18–64 years (28.6%) received the most specialty consultations (Table 2 and Additional file 1: Table S1).

In both older patients and patients 18–64 years, a higher MTS urgency was associated with a higher ED resource utilisation (Table 3). There was a stronger association between the MTS category and the number of diagnostic tests as well as multiple specialty consultations in the reference group than in older patients (Table 3).

### Hospitalisation

In total, 13.0% of the older patients were discharged home without a follow-up, 27.3% with a follow-up and 14 patients (0.2%) died on the ED (Table 2). The ED visits by older patients resulted in more hospital admissions than ED visits by patients 18–64 years (59.4% versus 24.5%, *p* < 0.001). In older patients the median hospital length of stay decreased significantly with a lower MTS urgency from 7.0 days (IQR 2–14) in category red to 5.0 days (IQR 2.0–10.0) in categories yellow and green (*p* = 0.012).

**Fig. 2:**
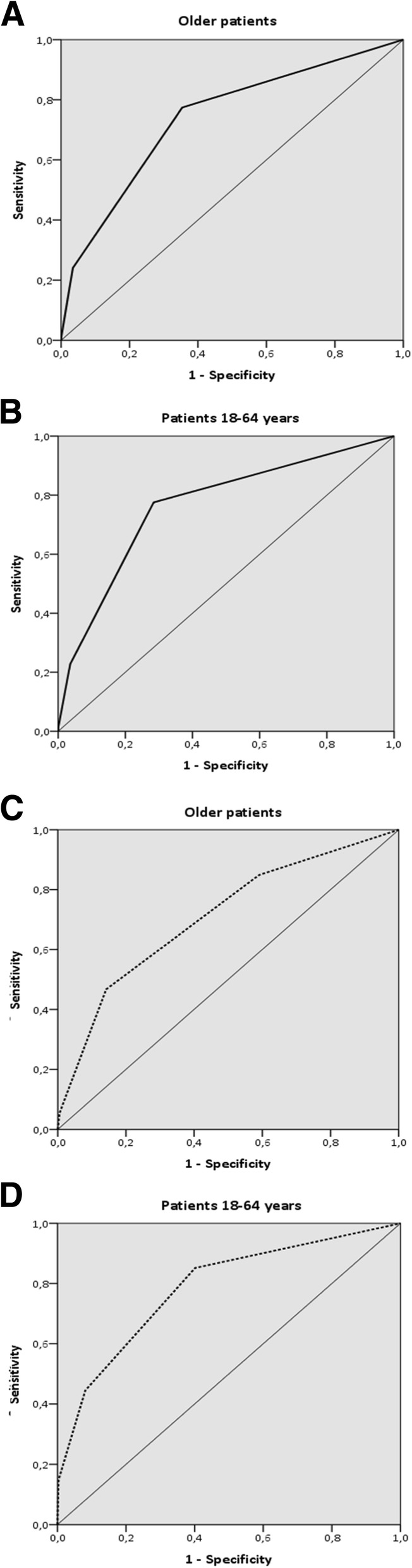
ROC for the MTS and hospitalisation and in-hospital mortality in older patients, and patients aged 18–64 years. 2A: ROC for the MTS and hospitalisation in older patients (AUC 0.74, 95% CI 0.73–0.75). 2B: ROC for the MTS and hospitalisation in patients 18–64 years (AUC 0.76, 95% CI 0.76–0.77). 2C: ROC for the MTS and in-hospital mortality in older patients (AUC 0.71, 95% CI 0.68–0.74). 2D: ROC for the MTS and in-hospital mortality in patients 18–64 years (AUC 0.79, 95% CI 0.72–0.85)

The risk of hospital admission following the ED visit was strongly associated with the MTS category in both older patients and patients 18–64 years. Older patients in the yellow category had a 4.8-fold (95% CI 4.3–5.3) higher risk of hospitalisation than the green category, in patients 18–64 years the risk increase in yellow versus the green category was significantly higher (OR 7.0, 95% CI 6.4–7.8) (Table 3). The AUC for the MTS and hospitalisation was 0.74 (95% CI 0.73–0.75) in older patients and 0.76 (95% CI 0.76–0.77) in patients 18–64 years (Fig. 2). The likelihood of admission to a high care unit was associated with MTS category as well in older patients (Table 2), with an AUC of 0.79 (95% CI 0.77–0.81).

### Mortality

In-hospital mortality was 7.5% in older patients compared with 1.7% in patients 18–64 years (*p* < 0.001). The mortality risk increased with increasing MTS category in older patients from 1.7% in the green category to 34% in the red category (Table 2).

Comparison of the in-hospital mortality in older patients with patients 18–64 years demonstrated similar associations with the MTS category (Table 3). The predictive ability of the MTS for in-hospital mortality was fair in older patients as well as patients 18–64 years with AUCs of 0.71 (95% CI 0.68–0.74) and 0.79 (95% CI 0.72–0.85) respectively (Fig. 2).

### Medical specialty

The majority of older patients assigned to the MTS category red and orange were treated by a medical specialty (85.2 and 84.9% respectively, *p* < 0.001) (Table 1). The mean age of older patients was comparable between surgical and medical specialties (76.9 years and 77.1 years, respectively, *p* = 0.259).

Overall, older surgical patients received fewer diagnostic tests and medical procedures on the ED (mean 1.2 and 1.1 respectively) than medical patients (mean 3.0 and 1.7 respectively, *p* < 0.001) (Table 4). The association between the MTS category and the number of diagnostic tests was comparable among older surgical patients and older medical patients (Table 4). In 11.7% of older surgical patients > 1 specialty consultations took place on the ED compared with 16.5% in older medical patients (*p* < 0.001). Overall, the median ED-LOS was considerably shorter in older surgical patients (111 min, IQR 71–158) compared with older medical patients (153 min, IQR 116–193, *p* < 0.001) (Table 4).

**Table 4:** ED resource utilisation, hospitalisation and in-hospital mortality per MTS category in older surgical and medical patients

Outcome measure	Group	MTS category – relative estimates with 95% confidence intervals			
		Red^‡^	Orange^‡^	Yellow^‡^	Absolute value in green reference category
		surgical: n = 9	surgical: *n* = 160	surgical: *n* = 1271	surgical: *n* = 1775
		medical: *n* = 52	medical: *n* = 903	medical: *n* = 1894	medical: *n* = 1044
ED-LOS (minutes, difference)^¶^	Surgical	23.4 (− 22.5–69.4)	61.8 (50.5–73.2)**	31.8 (26.7–36.8)**	Median 95 (59–140)
	Medical	−56.2 (− 73.6- -38.7)	−19.1 (− 24.7- -13.5)**	5.2 (0.4–9.9)	Median 158 (119–200)
Number of diagnostic tests (IDR)^^^	Surgical	3.6 (2.4–5.3)**	3.6 (3.3–4.0)**	2.2 (2.0–2.3)**	Mean 0.8 (0.9)
	Medical	1.5 (1.2–1.7)**	1.8 (1.7–1.9)**	1.4 (1.3–1.4)**	Mean 2.2 (1.4)
Number of medical procedure (IDR)^^^	Surgical	3.1 (2.1–4.7)**	3.1 (2.8–3.4)**	1.6 (1.5–1.7)**	Mean 0.8 (0.9)
	Medical	3.5 (3.0–4.1)**	2.5 (2.3–2.7)**	1.6 (1.5–1.7)**	Mean 1.0 (0.9)
Medication administered (OR)^±^	Surgical	4.0 (1.1–15.0)**	6.6 (4.7–9.4)**	3.9 (3.4–4.6)**	*N* = 423 (23.8%)
	Medical	4.8 (2.7–8.7)**	4.4 (3.7–5.4)**	1.8 (1.5–2.1)**	*N* = 312 (29.9%)
> 1 specialty consultations (OR)^±^	Surgical	–	9.0 (6.2–13.0)**	2.6 (2.1–3.3)**	*N* = 117 (6.6%)
	Medical	1.4 (0.7–3.1)	1.7 (1.4–2.3)**	1.8 (1.5–2.3)**	N = 117 (11.2%)
Hospitalisation (OR)^#±^	Surgical	–	22.9 (15.0–34.9)**	6.2 (5.2–7.3)**	*N* = 291 (16.4%)
	Medical	–	9.1 (6.8–12.4)**	2.6 (2.2–3.1)**	*N* = 660 (63.2%)
In-hospital mortality (OR)^# ±^	Surgical	50.4 (9.3–273.3)**	19.7 (8.8–44.3)**	4.1 (2.0–8.5)**	N = 10 (0.6%)
	Medical	15.7 (7.7–32.1)**	4.0 (2.7–5.9)**	1.4 (0.95–2.1)	*N* = 37 (3.5%)

*MTS* Manchester Triage System; *ED* Emergency Department; Surgical includes: general surgery, plastic surgery, urology, orthopaedics, otorhinolaryngology, ophthalmology, dermatology, oral surgery, gynaecology; Medical includes: internal medicine, pulmonology, cardiology, neurology, psychiatry, gastroenterology, rheumatology; *SD* standard deviation; *IQR* interquartile range; *ED-LOS* emergency department length of stay. ‡ = Comparison of MTS category in older surgical and medical patients with reference category, which is MTS category green; ^= analysed with Poisson regression, reporting incidence density ratios; ± = analysed with logistic regression, reporting odds ratio and 95% confidence intervals; ¶ = difference with MTS green in minutes, analysed with linear regression, reporting regression coefficients and 95% confidence intervals; # = patients who have died in the ED are excluded from the analyses; ** = *p* < 0.001

Hospital admission was more frequent in older medical patients (79.9%) than older surgical patients (35.0%) (Table 4). The predictive ability of the MTS for hospitalisation was better in older surgical patients than older medical patients (AUC 0.74, 95% CI 0.72–0.76 versus AUC 0.69, 95% CI 0.67–0.71 respectively). In total, 34 older surgical patients (1.1%) were admitted to a high care unit compared with 459 older medical patients (11.8%) (*p* < 0.001). The AUC for the MTS and admission to a high care unit was 0.84 (95% CI 0.77–0.92) in older surgical patients, and 0.73 (95% CI 0.70–0.75) in older medical patients.

Overall, in-hospital mortality was 5.1% in older surgical patients, and 8.4% in older medical patients (*p* < 0.001). The in-hospital mortality in older medical patients was higher in every MTS category as compared with older surgical patients (p < 0.001) (Table 4). The difference in in-hospital mortality in older medical patients assigned to category yellow relative to category green was not statistically different (OR 1.4, 95% CI 0.95–2.1) (Table 4). The AUC for the MTS and in-hospital mortality was 0.74 (95% CI 0.68–0.81) in older surgical patients, and 0.66 (95% CI 0.62–0.69) in older medical patients.

## Discussion

In our retrospective cohort, we have demonstrated that the MTS is associated with the ED resource utilisation, risk of hospitalisation and in-hospital mortality in older patients (≥ 65 years old). However, the performance of the MTS as a predictor of ED resource utilisation and in-hospital mortality in older patients was not as good as in patients aged 18–64 years (i.e. reference population). Stratifying the performance of the MTS by specialty revealed a better predictive ability of the MTS for hospitalisation and in-hospital mortality in older surgical patients than older medical patients.

Research on the validity of triage tools is complicated by the lack of a gold standard for true patient acuity. We based the performance of the MTS on its ability to predict hospitalisation and in-hospital mortality as a surrogate for acuity. In addition, we used the ED resource utilisation and the ED-LOS as a proxy for workload. These outcome measures are clinically relevant and correspond to the objective of the MTS, which is to prioritise patients based on clinical acuity in a setting with limited resources and persistent time pressure [13].

Our study confirms previous findings of worse performance of the MTS in older patients compared with younger patients [21, 22]. These results are also consistent with previous studies on the validity of another triage system, the Emergency Severity Index (ESI), in older patients, which demonstrated that older patients are at risk of undertriage [7, 27]. These findings emphasise that older patients represent a special population on the ED with distinct care needs, similar to the paediatric population in which the performance of the MTS has been investigated [15, 16]. However, it is possible that increased complexity due to multi-morbidity could explain the lower performance of the MTS in older patients rather than chronological age [28]. Multi-morbidity, which is more common in older patients, often results in a challenging and time consuming triage process [29, 30]. Additionally, acute medical illnesses in older patients might be masked by an atypical presentation, such as generalised weakness or altered mental status, possibly contributing to a higher risk of undertriage [7, 21, 22, 29, 31]. Therefore, it is imperative that ED personnel are aware of the weaker performance of the MTS in older patients in order to prevent possible risks associated with inadequate triage in older ED patients, especially in medical patients.

Consistent with previous studies, the ED-LOS increased across MTS categories green to orange, and was shortest in category red in older patients [19]. The association between the ED-LOS and the MTS was better in the older patients. However, the ED resource utilisation appeared to be less evidently associated to MTS category in older patients than in patients aged 18–64 years. The demand on resources by older patients at the ED is high, even in the green MTS category. Furthermore, over one third of older patients in category green were hospitalised following the ED visit. These findings might elucidate the complexity of emergency care evaluation of older patients, as well as their impact on emergency care processes.

The predictive ability of the MTS for hospitalisation in our cohort of older patients (AUC 0.74) was fair, which is consistent with a previous study in the general population [19]. The admission rate in our older population was considerably higher (60%) than the 40–46% seen in other studies that focus solely on older patients [27, 32], which might be caused by the high admission rate in non-urgent patients, such as category green (33.8%) compared with other studies [7, 30]. This could be explained by the difference in the health care system. The emergency care system in the Netherlands cooperates intensely with GPs, who offer around the clock emergency care [24], which may result in referral of the more severely ill patients.

In both older patients and patients aged 18–64 years, the in-hospital mortality increased with an increasing MTS urgency, which is consistent with existing literature [19, 33]. However, we found a difference in the predictability of the MTS for in-hospital mortality in older patients compared with patients aged 18–64 years (AUC 0.71 versus 0.79). A possible explanation is the relative higher in-hospital mortality rate in older patients in non-urgent categories yellow and green (3.9 and 1.7%) compared with patients aged 18–64 years (0.5 and 0.1%), which might be a reflection of advanced age, higher comorbidity level or more severe unrecognised illness in older patients [11, 29, 34].

Our study demonstrated a difference in the performance of the MTS in older surgical patients compared with older medical patients. The ED resource utilisation was higher in older medical patients compared with surgical patients. Furthermore, the admission rate in medical patients was considerably higher than in surgical patients (80% versus 35%), which might be elucidated by a higher percentage of elective admission for surgical patients. Moreover, in-hospital mortality in older patients categorised yellow and green was significantly higher in medical specialties than surgical specialties (4.9% versus 2.3 and 3.5% versus 0.6%, respectively). In contrast with previous research, the MTS performed better in older surgical patients than medical patients regarding the number of diagnostic tests performed, and the ED-LOS [14]. In addition, the MTS appeared to more accurately predict hospitalisation (AUC 0.74 versus 0.69), admission to a high care unit (AUC 0.84 versus 0.73), and in-hospital mortality (AUC 0.74 versus 0.66) in older surgical patients than older medical patients. These findings might be explained by a higher complexity of older medical patients than surgical patients and more severe underlying illness requiring more resources, resulting in a longer ED-LOS and a higher admission rate [33].

### Limitations

Our results may have been influenced by several limitations. Firstly, owing to the retrospective observational design of the study, there is a risk of bias. The perception of acuity may differ from the real urgency of a patient’s condition, which is difficult to identify based on administrative data. Secondly, our findings may be less generalisable to other hospitals and countries, because of the single-centre setting and distinct health care organisation in the Netherlands. Therefore, the organisation of emergency care in other countries should be considered when interpreting our findings. The majority of patients presenting with emergency cardiac and gynecological complaints were not included in this study, therefore our results may be less applicable to these patients. Thirdly, the impact of comorbidity on the difference performance of the MTS in older and younger patients was not taken into account in our study. Fourthly, only including admission to a high care unit or the need for immediate lifesaving intervention in the assessment of the performance of the MTS might be insufficient in older patients to identity actual acuity. This might result in an overestimation of the performance of the MTS, because of possible confounding by treatment constraints, such as a do not resuscitate order or a no ICU admission order. Last, the chance of a type 1 error was larger than 0.05 due to multiple testing.

## Conclusion

In our retrospective cohort, the MTS appeared to perform worse in older patients (≥ 65 years old) as compared with younger patients (18–64 years old). Although, the MTS was associated with ED resource use, ED-LOS, hospitalisation and in-hospital mortality in older patients, the predictive ability of the MTS for in-hospital mortality was worse in older patients than in patients aged 18–64 years. The MTS demonstrated a better performance in older surgical patients than older medical patients regarding hospitalisation and in-hospital mortality. These findings emphasise the need for an increased awareness of the higher risk of adverse outcome in older emergency department patients, particularly, in older medical patients.

## Additional file

Additional file 1: **Table S1.** Characteristics of emergency department visits by patients aged 18–64 years per MTS category. Comparison of the following characteristics in patients aged 18–64 years old per MTS category: age, mode of referral, specialty, median ED-LOS, ED resources utilisation, disposition and in-hospital mortality (DOCX 17 kb)

## Abbreviations

ANOVA: Analysis of variance
AUC: Area under the curve
CCU: Cardiac care unit
CI: Confidence interval
ED: Emergency department
ED-LOS: Emergency department length of stay
ESI: Emergency Severity Index
GP: General Practitioner
ICU: Intensive care unit
IDR: Incidence density ratio
IQR: Interquartile range
LWBS: Left without been seen by a physician
MCU: Medium care unit
MTS: Manchester Triage System
OR: Odds ratio
ROC: Receiver operating characteristics
SCU: Stroke care unit
SD: Standard deviation
