# Supplementary material for: Performance of the Manchester triage system in older emergency department patients: a retrospective cohort study
Source: BMC Emerg Med. 2019 Jan 7;19:3. doi: 10.1186/s12873-018-0217-y (PMC6322327; doi:10.1186/s12873-018-0217-y)
Supplement: Supplementary file 1 — Table S1. Characteristics of emergency department visits by patients aged 18–64 years per MTS category. Comparison of the following characteristics in patients aged 18–64 years old per MTS category: age, mode of referral, specialty, median ED-LOS, ED resources utilisation, disposition and in-hospital mortality (DOCX 17 kb) [file 12873_2018_217_MOESM1_ESM.docx]

**Table S1 Characteristics of emergency department visits by patients aged 18-64 years per MTS category.**

|  | **MTS category** | | | |
| --- | --- | --- | --- | --- |
|  | **Red**  **(n =56)** | **Orange**  **(n = 1,084)** | **Yellow**  **(n = 4,412)** | **Green**  **(n = 8,215)** |
| Mean age in years (SD)** | 46.3 (13.8) | 43.9 (14.3) | 43.2 (13.7) | 39.3 (14.1) |
| Male participants (%)** | 35 (62.5%) | 568 (54.1%) | 2,223 (50.4%) | 4,806 (58.5%) |
| Mode of referral (%)** |  |  |  |  |
| - General practitioner | 17 (30.9%) | 367 (35.2%) | 1,757 (42.2%) | 1,791 (23.0%) |
| - Self-referral | 4 (7.3%) | 222 (21.3%) | 1,458 (35.0%) | 4,624 (59.4%) |
| - Ambulance | 33 (60.0%) | 396 (37.9%) | 579 (13.9%) | 277 (3.6%) |
| - Medical specialist | 1 (1.8%) | 59 (5.7%) | 373 (9.0%) | 1,094 (14.1%) |
| Specialty (%)** |  |  |  |  |
| - Surgical | 12 (21.4%) | 426 (39.3%) | 2,398 (54.4%) | 6,863 (83.5%) |
| - Medical | 44 (78.6%) | 658 (60.7%) | 2,014 (45.6%) | 1,352 (16.5%) |
| Median ED-LOS in minutes (IQR)** | 103 (70-170) | 127 (94-170) | 126 (87-171) | 80 (50-124) |
| ED resource utilisation |  |  |  |  |
| - Mean number of diagnostic tests (SD)** | 3.3 (1.9) | 2.7 (1.7) | 1.7 (1.4) | 0.7 (0.9) |
| - Mean number of medical procedures (SD)** | 3.7 (1.6) | 2.0 (1.2) | 1.0 (1.0) | 0.8 (0.8) |
| - Medication administered (%)** | 48 (85.7%) | 644 (59.4%) | 2,269 (51.4%) | 1,800 (21.9%) |
| - >1 specialty consultations on ED (%)** | 16 (28.6%) | 213 (19.6%) | 504 (11.4%) | 242 (2.9%) |
| Disposition (%)** |  |  |  |  |
| - Discharge home | 2 (3.6%) | 190 (17.5%) | 938 (21.3%) | 3,003 (36.6%) |
| - Discharge home + follow-up | - | 161 (14.9%) | 1,629 (36.9%) | 4,437 (54.0%) |
| - Admission acute medical unit | 10 (17.9%) | 490 (45.2%) | 1,581 (35.8%) | 686 (8.4%) |
| - Admission other hospital ward | 39 (69.6%) | 239 (22.0%) | 258 (5.8%) | 71 (0.9%) |
| - LWBS | - | 3 (0.3%) | 6 (0.1%) | 17 (0.2%) |
| - Died in ED | 5 (8.9%) | 1 (0.1%) | - | - |
| In-hospital mortality^#^ (%)** | 10 (19.6%) | 17 (1.6%) | 22 (0.5%) | 8 (0.1%) |
| MTS = Manchester Triage System; SD = Standard Deviation; ED = Emergency Department; Surgical includes: general surgery, plastic surgery, urology, orthopaedics, otorhinolaryngology, ophthalmology, dermatology, oral surgery, gynaecology; Medical includes: internal medicine, pulmonology, cardiology, neurology, psychiatry, gastroenterology, rheumatology; ED-LOS = emergency department length of stay; IQR = interquartile range. P-values were calculated using ANOVA, Kruskal-Wallis test and Chi-square test; ** = p < 0.001; # = patients who have died in the ED are excluded from this analysis. | | | | |
